# Integrating dermatologists in primary care: impact on delays, patient and professional experiences

**DOI:** 10.1186/s12913-024-11923-y

**Published:** 2024-11-20

**Authors:** Maria Lovén, Amanda Eklund, Laura Huilaja, Markus Paananen, Paulus Torkki

**Affiliations:** 1 Department of Public Health, The University of Helsinki, Tukholmankatu 8, 00014, Helsingin yliopisto, Finland; 2 Mehiläinen Länsi-Pohja, Kauppakatu 25, 4. Krs, 94100 Kemi, Finland; 3 Department Industrial Engineering and Management, The Aalto University, Aalto, P.O. Box 11000, 00076 Espoo, Finland; 4 Department of Dermatology and Medical Research Center, Oulu University Hospital, Kajaanintie 50, 90220 Oulu, Finland; 5 Research Unit of Clinical Medicine, University of Oulu, P.O.Box 8000, 90014 Oulu, Finland; 6 The University of Oulu, Aapistie 5a, 90220 Oulu, Finland; 7 Western Uusimaa Wellbeing Services County, PL 33, 02033 Länsi-Uudenmaan Hyvinvointialue, Finland; 8 Department of Public Health, The University of Helsinki, Tukholmankatu 8, 00014 Helsingin Yliopisto, Finland

**Keywords:** Integrated health care systems, Primary healthcare, General practice, Dermatologists, Delay, Patient experience, PREM, PEI, Health personnel, Professional satisfaction

## Abstract

**Background:**

Primary healthcare centres are burdened by the management of patients with skin conditions, while general practitioners might lack the expertise to assess skin changes accurately. The traditional care chain for skin findings is a multistage process that can cause delayed diagnosis and treatment, distressing the patient.

This study aimed to determine whether adding a dermatologist to the primary care team would streamline the care pathway of patients with skin conditions, while examining levels of satisfaction among patients and healthcare professionals.

**Methods:**

A quasi-experimental multicentre study was conducted in three primary health centres in Finland. A dermatologist was integrated into two of the centres (intervention) but not the third (control). Data on timing of diagnosis and treatment and number of contacts were collected from records and analysed per care path. The Patient Enablement Instrument (PEI) and Net Promoter Score (NPS) were used to measure the patient’s experience of the appointment. NPS and professional satisfaction queries were used to measure professional satisfaction.

**Results:**

In total 186 intervention and 176 control patients were included, with 38 primary care professionals. Compared with the control group, the intervention group showed a significantly shorter time to confirmed diagnosis and to treatment start (25 vs. 49 days, *p* < 0.001), with a higher proportion (49% vs. 27%, *p* < 0.001) receiving immediate treatment in the primary care setting. Patients in the intervention group required fewer visits. Patient experience by PEI and NPS scores were higher in the intervention group (*p* ≤ 0.022 for each). Satisfaction levels among professionals in both groups were higher after the intervention than before, although the NPS score did not improve significantly in the control group. Almost all professionals advocated for the continuation of the integrated care pathway.

**Conclusions:**

The integration of dermatologists into the primary care streamlined the management of skin conditions from diagnosis to treatment, while improving the experiences of both patients and healthcare professionals. This integrated care path is beneficial for the management of patients with skin findings in primary care.

**Supplementary Information:**

The online version contains supplementary material available at 10.1186/s12913-024-11923-y.

## Background

Skin diseases were the eighth most common diagnosis made at public primary care doctors appointments in Finland in 2023 [1]. Two-thirds of middle-aged individuals and three-quarters of those over 70 have a skin disease, with nearly half requiring further treatment [2, 3]. According to the Finnish cancer register, the incidence of skin cancer has increased nine-fold since the 1960s [4].

Patients with skin lesions often require a biopsy, which imposes a burden on the healthcare system [5]. The potential malignancy associated with lesions causes distress to the patient [6]. Many cases of skin cancer remain undetected at general practitioner (GP) appointments [7, 8]. Delays correlate with reduced survival rates [9, 10]. Also basal cell carcinoma and non-malign dermatological diseases, like Hidradenitis suppurativa are more likely to manifest with an aggressive clinical course when detected and treated later in the disease course [11–14].

Evidence from other specialties suggests that the presence of hospital specialists in primary care clinics could reduce delays and improve patient experience [15–17], although results have been inconsistent with regard to dermatology [18–20]. Melanoma detection could be enhanced with fewer biopsies [21]. Improvements in the patient experience are associated with higher levels of adherence to prevention and treatment, better clinical outcomes and lower healthcare utilization [22–26].

The objective of the present study was to investigate whether the early involvement of a dermatologist in the primary care of patients presenting with skin changes would affect diagnostic and treatment timelines, and to evaluate the impact of the integrated care pathway on the experiences of patients and the professionals who treat them.

## Methods

### Study design

This was a quasi-experimental multicentre intervention study with a follow-up of one year. In the intervention group, patients presenting with skin issues were seen by a dermatologist in primary care, rather than a GP. Patients in the control group (standard care) were initially seen by a GP.

The study took place at the health centres of Tornio and Keminmaa (interventions) and Kemi (Control), in northern Finland, where the health care system is publicly funded and operates on a capitation basis. Each health centre provides primary healthcare services to all the residents in its designated catchment area (Table 1).

**Table 1:** Health centres

Health centre	Tornio	Keminmaa	Kemi
Status	Intervention	Intervention	Control
Total Population	21 467	7 984	20 437
Study patients	116	70	176
Nurses	16	6	14
General practitioners	8	4.5^a^	8

^a^Resources as Full-time equivalent

Eligible patients included individuals aged at least 18 years who visited one of the study centres with a skin finding which needed to be assessed between March 2021 and April 2022. Findings that indicated a possible need for biopsy or surgery, such as suspect melanocytic naevi or lumps, were prioritized. For inclusion, patients had to be able and willing to give written informed consent. Patients already in the process of treatment of the same skin finding were excluded from the study.

All general practitioners and nurses working regularly at the study health centres were invited to attend the study; participation was voluntary and anonymous.

### Intervention

Dermatologists were introduced into the primary care setting in the Tornio and Keminmaa centres; while the Kemi centre continued with standard primary care practice. Participants were assigned to the ‘intervention’ or ‘control’ group according to which centre they attended. Participants in the intervention group underwent dermatological assessments in the primary care setting without the GP making a referral to the dermatologist. The protocol enabled nurses to schedule appointments directly with a dermatologist, who was equipped with a dermoscope and liquid nitrogen for cryotherapy. The dermatologist was supported by two nurses who were also responsible for disseminating both oral and written information about the study and for obtaining patient consents. Initial information regarding the study was typically provided to patients during their first contact with healthcare services concerning their dermatological issue, often through a telephone conversation.

Conversely, the control group in Kemi adhered to standard primary care practices, where general practitioners conducted the initial examinations. A nurse was tasked with informing these patients about the study and gathering consent forms.

Diagnoses were confirmed through dermoscopy or histopathological examination (pathological-anatomic diagnosis [PAD]), with the latter being performed at the Central Hospital in Kemi by a certified pathologist.

### Data collection

Immediately following their research appointment with a doctor, participants responded on a pseudonymized paper questionnaire to the Patient Enablement Instrument question two (PEI Q2): “As a result of your visit to the doctor today, do you feel you are able to cope with your illness…” with possible answers ‘Much better’; ‘Better’; or ‘Same or Less’ (Additional file 1) [27]. The PEI Q2 is an established patient-reported outcome measure that reflects the quality of appointments with GPs [27]. On the same questionnaire, patients provided a grade on the likelihood that they would recommend the service of the healthcare provider they had just visited (0 = ‘very unlikely’ to 10 = ‘very likely’), from which a Net Promoter Score (NPS) [28, 29] could later be derived. In the calculation of NPS categories, participants returning grades 0–6 were considered as ‘Detractors’, 7–8 ‘Passives’ and 9–10 ‘Promoters’. The NPS was calculated as (number of Promoters—number of Detractors) / (Total number of respondents).

For each patient, clinical information was retrieved from their electronic record (OMNI by CGI) [30]. This included information on relevant diagnosis, multimorbidity, previous skin diseases and smoking status. Timestamped information on the care pathway subsequent to their study-related diagnosis was also collected. Multimorbidity was defined as the presence of at least three long-term illness requiring regular care or monitoring. Long-term refers to a period of at least six months [31].

The healthcare professionals filled semi-structured anonymous paper questionnaires regarding their working experience pre- and post-intervention (Additional files 2 and 3). Responses on satisfaction were given on a 5-point Likert scale, which ranged from 'very unsatisfied' to 'very satisfied', and as an NPS grade.

### Study outcomes

The primary outcome was time from first contact to the start of treatment. The secondary outcomes were time from first contact to first diagnosis and to confirmed diagnosis, delay from the doctor visit to the start of treatment, number of visits per care pathway, the proportion of patients treated in primary care and the proportion of patients treated at all, the distribution of responses to the PEI Q2 and NPS derived from the responses to the patient questionnaire. For measurement of healthcare professional experience, the outcomes were satisfaction as reported on the Likert scale and NPS, change in workload, learning of new skills, patient utility and professional’s willingness to adopt the new care pathway.

### Sample size and statistical analysis

The ClinCalc.com website was used to calculate sample size [32]. Power calculations revealed that 140 patients were needed per group to obtain a power of 80% (Fisher’s exact, two‐sided, α = 0·05).

The normality of data distributions was assessed with the Shapiro-Wilkins test. Categorical variables were tested with Pearson’s Chi-Squared test or Fisher’s Exact test and continuous variables with the Mann–Whitney U test. The logistic regression model was constructed for the categorical variables and linear regression was applied to the continuous variables to estimate the associations between the intervention and dependent variables. Crude and adjusted odds ratios, confidence intervals, p-values and Akaike information criterion (AIC) [33] were determined. Age, sex, smoking status, previous skin diseases and multimorbidity were recognized as potential confounders and were considered in the statistical analysis for all measured parameters. Additionally, a delay from the first contact to doctor visit was included as a confounding factor in the regression model. Missing data were reported and were not imputed in the analyses.

The R studio software package [34] version 4.2.2 was used for the statistical analysis of the research data. A p value n ≤ 0.05 was considered statistically significant.

## Results

Evaluable data were available for a total of 362 patients, 186 patients in the intervention group and 176 in the control group. A history of skin disease was more common in the intervention group but other baseline characteristics were comparable (Table 2).

**Table 2:** Baseline characteristics of the population

	**Intervention**	**Control**	***p* value**
	*n* = 186	*n* = 176	
Sex			0.558
Male	57 (30.6%)	59 (33.5%)	
Female	129 (69.4%)	117 (66.5%)	
Age			0.789
Median (IQR)	66 (47, 74)	65 (45, 73)	
Range	19 – 93	18 – 96	
Previous skin disease			0.005
No	106 (57.6%)	123 (71.9%)	
Yes	78 (42.4%)	48 (28.1%)	
Multimorbidity^a^			0.582
No	93 (50.3%)	92 (53.2%)	
Yes	92 (49.7%)	81 (46.8%)	
Smoking			0.118
No	105 (68.2%)	90 (57.0%)	
Ex-smoker^b^	33 (21.4%)	44 (27.8%)	
Yes	16 (10.4%)	24 (15.2%)	

*IQR* interquartile range

^a^Patient has ≥ 3 illnesses for which they receive regular care or are monitored by the healthcare professional for a period of ≥ 6 months

^b^Patient has smoked, but quit according to patient record

The median times from first contact to treatment initiation, to first diagnosis and to diagnosis confirmation were all shorter in the intervention group than in the control group, as was the median time from first contact to the doctor visit (Table 3). Except for the time to first diagnosis, the group differences remained significant after adjustment for detected confounding factors.

**Table 3:** Process outcomes

	**Intervention**	**Control**	**Total**	
	*n* = 186	*n* = 176	Total *n* = 362	*p* value
**Primary outcome**				
Time from first contact to treatment start				
n	94	58	152	
Median (Q1, Q3)^a^	25.0 (13. 3, 44.5)	49.5 (9.0, 87.5)	30.5 (12.0, 57.3)	0.005
Range	0.0 – 119.0	0.0 – 325.0	0.0 – 325.0	
**Secondary outcomes**				
Time from first contact to first diagnosis				
n	184	172	356	
Median (Q1, Q3)	25.0 (12.0, 39.0)	31.0 (7.0, 56.0)	27.0 (10.8, 46.3)	0.028
Range	0.0 – 154.0	0.0 – 280.0	0.0 – 280.0	
Time from first contact to first confirmed diagnosis				
n	185	90	275	
Median (Q1, Q3)	29.0 (17.0, 50.0)	56.0 (38.0, 73.0)	38.0 (21.5, 57.5)	< 0.001
Range	0.0 – 154.0	0.0 – 280.0	0.0 – 280.0	
Time from first contact to doctor visit				
n	186	176	362	
Median (Q1, Q3)	25.0 (14.3, 43.8)	37.00 (21.8, 60.5)	30.0 (15.0, 52.0)	< 0.001
Range	0.0 – 154.0	0.0 – 280.0	0.0 – 280.0	
Time from first doctor visit to treatment start				
n	96	59	155	
Median (Q1, Q3)	0.0 (0.0, 0.0)	0.0 (0.0, 14.0)	0.0 (0.0, 0.0)	0.003
Range	0.0 – 114.0	0.0 – 262.0	0.0 – 262.0	
Number of contacts during the care path				
n	186	176	347	
Median (Q1, Q3)	3.0 (2.0, 10.4)	5.00 (2.0, 17.0)	4.0 (2.0, 14.0)	< 0.001
Range	2.0 – 12.0	2.0 – 22.0	0.0 – 22.0	
	N of patients (%)	N of patients (%)		
Treatment started in Primary care				< 0.001
No	94 (50.5%)	129 (73.3%)	223 (61.6%)	
Yes	92 (49.5%)	47 (26.7%)	139 (38.4%)	
Treatment required and started at all				< 0.001
No	90 (48.4%)	117 (66.5%)	207 (57.2%)	
Yes	96 (51.6%)	59 (33.5%)	155 (42.8%)	

^a^Q1 Quartile 1; Q3: Quartile 3

The median number of contacts per care pathway was lower in the intervention group (3 vs. 5, *p* < 0.001). Treatment for skin conditions was received by 51.6% and 33.5% (*p* < 0.001) of patients in the intervention and control groups, respectively. In the intervention group, 49.5% started treatment in the primary care setting, compared with 26.7% in the control group (*p* < 0.001). These differences between the groups remained statistically significant after adjusting for age, sex, smoking, multimorbidity and previous skin diseases. Results of the linear regression (Table S1 and S2) and logistic regression (Table S3) are presented in the Additional file 4.

**Fig. 1:**
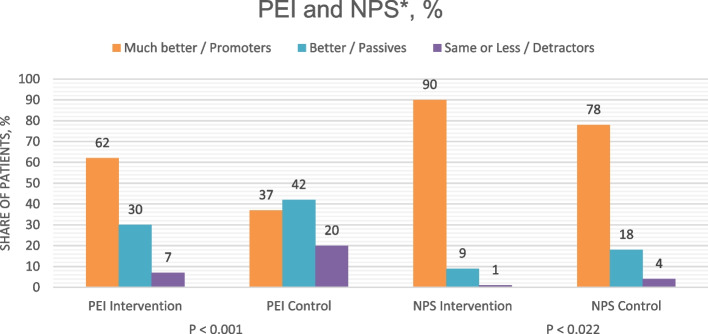
Patient enablement instrument, question 2 (PEI Q2) and Net Promoter Score (NPS) answer distribution. *Patient enablement instrument, question 2 (PEI Q2) and Net Promoter Score (NPS) questionnaire answer distributions directly after the doctor visit. A PEI Q2 is:“As a result of your visit to the doctor today, do you feel you are able to cope with your illness…” with possible answers ‘Much better’; ‘Better’; or ‘Same or Less’. In the calculation of NPS, participants giving responses of 0–6 were considered as ‘Detractors’, 7–8 ‘Passives’ and 9–10 ‘Promoters. The NPS was calculated as (number of Promoters—number of Detractors) / (Total number of respondents)

Patients’ ability to cope with their illness after a doctor's visit (PEI Q2) was better in the intervention group: 62.4% answered 'much better' and 30.3% ‘better’, compared with 37.2% and 42.2% in the control group (Table 4, Fig. 1). After adjusting, the effect of the intervention remained significant for ‘much better’ (*p* < 0.005) (Additional file 4, Table S4).

**Table 4:** Patient experience

**Patient experience**	**Intervention**	**Control**	***p*-value**
Response to Patient Enablement Instrument, question 2^a^			< 0.001
Much better	111 (62.4%)	64 (37.2%)	
Better	54 (30.3%)	73 (42.4%)	
Same or less	13 (7.3%)	35 (20.3%)	
Missing	8	4	
Net Promoter score (NPS)			0.022
Same or less	2 (1.3%)	4 (3.9%)	
Passives	14 (8.8%)	19 (18.4%)	
Promoters	143 (89.9%)	80 (77.7%)	
Missing	27	73	
NPS Total	88.70%	73.80%	

^a^“As a result of your visit to the doctor today, do you feel you are able to cope with your illness…”

NPS distribution was significantly more favorable in the intervention group (*p* = 0.02), showing summary values of 88.7% vs. 73.8 (Table 4, Fig. 1). After adjusting, the intervention effect remained statistically significant for ‘Promoters’ (*p* = 0.015), but not for Detractors (*p* = 0.89). (Additional file 4, Table S5).

### Professional satisfaction

In the intervention health centres, professionals’ Likert scale satisfaction and NPS with the care pathway improved after the intervention. The satisfaction grading of the control centre also increased after the intervention, but without a significant change in NPS (Table 5). The intervention and control groups did not differ at baseline or post-intervention in terms of the healthcare professionals’ education, working experience, satisfaction with the care pathway, or NPS distribution. (Additional File 4, Table S6 and S7).

**Table 5:** Professional satisfaction measures pre- and post-intervention

	**Intervention group**			**Control group**		
	**Pre**	**Post**	***p* value**	**Pre**	**Post**	***p* value**
	n (%)	n (%)		n (%)	n (%)	
n	24	28		14	9	
Education of the professional			0.575			0.436
Specialist	4 (16.7%)	3 (10.7%)		0 (0.0%)	0 (0.0%)	
Licentiate	5 (20.8%)	9 (32.1%)		2 (14.3%)	3 (33.3%)	
Medical student	0 (0.0%)	0 (0.0%)		1 (7.1%)	0 (0.0%)	
Nurse	15 (62.5%)	15 (53.6%)		11 (78.6%)	6 (66.7%)	
Other	0 (0.0%)	1 (3.6%)		0 (0.0%)	0 (0.0%)	
Working experience			0.064			0.495
< 3 months	0 (0.0%)	2 (7.1%)		1 (7.1%)	0 (0.0%)	
3–9 months	1 (4.2%)	6 (21.4%)		1 (7.1%)	0 (0.0%)	
> 9 months	23 (95.8%)	20 (71.4%)		12 (85.7%)	9 (100.0%)	
Satisfaction with care pathway			< 0.001			0.018
Very satisfied	2 (8.7%)	20 (71.4%)		1 (7.7%)	5 (55.6%)	
Satisfied	8 (34.8%)	4 (14.3%)		7 (53.9%)	4 (44.4%)	
Neutral	7 (30.4%)	4 (14.3%)		5 (38.5%)	0 (0.0%)	
Unsatisfied	5 (21.7%)	0 (0.0%)		0 (0.0%)	0 (0.0%)	
Very unsatisfied	1 (4.4%)	0 (0.0%)		0 (0.0%)	0 (0.0%)	
Net Promoter Score NPS			< 0.001			0.102
Detractors	9 (40.9%)	0 (0.0%)		1 (10.0%)	0 (0.0%)	
Passives	11 (50.0%)	5 (17.7%)		6 (60.0%)	2 (22.2%)	
Promoters	2 (9.1%)	23 (82.1%)		3 (30.0%)	7 (77.8%)	
NPS Total	- 31.8%	82.1%		20.0%	77.8%	
**Post questionnaire only**						
Managing a patient with a skin disorder is:						
Easier		21 (80.8%)			7 (77.8%)	
More complicated		1 (3.8%)			1 (11.1%)	
No different		4 (15.4%)			1 (11.1%)	
The new care pathway reduces workload						
Yes		15 (60.0%)			6 (66.7%)	
No		10 (40.0%)			3 (33.3%)	
My ability to treat skin conditions has improved						
A lot		3 (11.5%)			1 (11.1%)	
Somewhat		11 (42.3%)			4 (44.4%)	
Not at all		12 (46.2%)			4 (44.4%)	
The new care pathway is more beneficial to the patient than the previous model						
Yes		26 (100.0%)			9 (100.0%)	
No		0 (0.0%)			0 (0.0%)	
I would like to continue with the new care pathway						
Yes		25 (96.1%)			9 (100.0%)	
No		1 (3.9%)			0 (0.0%)	

In the post-intervention questionnaire, around 80% of professionals in each group opined that management of patients with skin findings was easier after the intervention, and at least 60% reported that the new care pathway reduced their workload. Over half in each group reported having improved their skill level in the treatment of skin disorders during the intervention. All the professionals agreed that the new care path was more beneficial to the patient than the previous model, and, aside from one professional, all advocated for its adoption as a standard care path in future (Table 5).

**Fig. 2:**
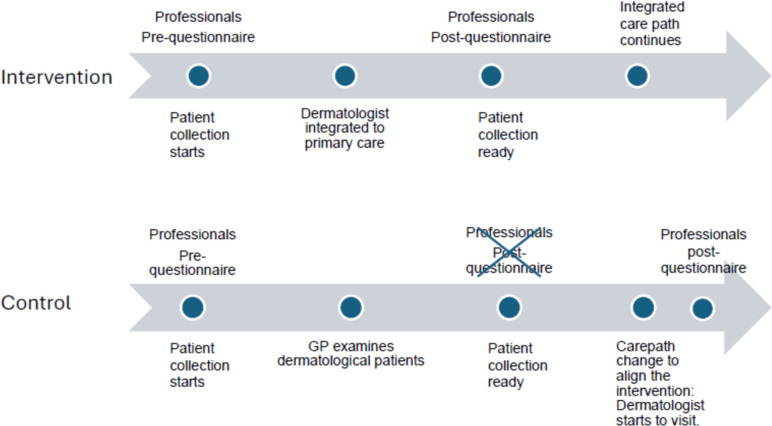
Data Collection

Contrary to the original study design, staff at the control healthcare centre failed to return their professional satisfaction post-questionnaires in the required time frame. After the end of the study patient collection, this control health centre then also adopted a carepath way and working model based on the study intervention as everyday practice. The staff at the Kemi health centre were then sent the ‘intervention’ version of the professional post-questionnaire, which they subsequently completed and returned – this should be noted as a breach of study protocol, and the pertinent results viewed with caution (Fig. 2).

## Discussion

We found that the presence of a dermatologist in primary care led to reduced delay in confirmed diagnosis and in treatment initiation in a primary care population. The number of contacts per patient in the intervention care pathway decreased significantly. The patient experience, as measured by the PEI Q2 and NPS, improved with the intervention. Levels of professional satisfaction at the intervention health centres were greater with the integrated than with the pre-intervention standard care pathway. This was also true of the control centre, although this finding was made outside the planned study framework, as described elsewhere in this paper. All healthcare professionals involved in our study agreed that the new care pathway was beneficial for patients, and almost all expressed a desire for its continuation.

The result considering delays are in line with the previous studies in several fields, most of which found that waiting times for specialist appointments were shorter when the specialist was based in primary care than when the first specialist visit was provided in the outpatient setting [16, 18, 19, 35–38]. However, as far as we know, ours is the first study to report changes in times to diagnosis and treatment initiation with an integrated care pathway. The designs of the previous studies also differed from ours and their specialists performed a more consultative role rather than partly replacing the GP as ours did.

Patient-reported experience measures (mainly unstandardized) tend to be higher in patients receiving specialist treatment in outreach clinics than those in hospital-based care [16, 17, 36, 37, 39], although some studies yielded contradictory results [18, 19]. A previous study of patients with malignant melanoma diagnosis found that the odds of patient dissatisfaction grew 3.5-fold with each additional visit required, unaffected by diagnosis delay, biopsy performer, or patient demographics [40]. Besides the treatment provided by the specialist, fewer contacts required may have improved patient satisfaction also in our study. A Swedish study, perhaps surprisingly, found no correlation between delay in melanoma diagnosis and patient satisfaction [41].

Professionals’ desire to continue with the integrated care pathway echoes findings of previous studies [35, 42]. The majority of professionals reported that the management of patients was easier with the intervention care pathway, 60% reported their workload had reduced (70% of them nurses). In line with previous studies, [43–45] over half of the primary care personnel, mostly nurses, reported having improved their ability to treat skin conditions during the intervention. Previous research on specialist outreach noted limited GP-specialist interaction opportunities; however those who did interact reported a benefit, which was also the case in our intervention [18]. Major advantages of integration, reported in this and previous studies, were overall improvements in communication, collaboration, trust, reciprocity and mutual respect between primary carers and hospital professionals [35, 43, 44, 46]. Professionals also have concisely described the model as “worthwhile” [35, 47].

We also interviewed the Consulting Dermatologist and the Chief Dermatologist at local Central Hospital where patients were referred if needed. They concurred that the intervention appeared beneficial and noted that referrals were more appropriate, concentrating severe cases at the hospital. They rated their satisfaction with the new integrated care pathway for NPS 8 and 10 and Likert scale grades of ‘satisfied’ to ‘very satisfied’. However, the Chief Dermatologist raised concerns about the potential for loss of clinical time and reduced specialist availability at the hospital if specialists were constantly required to travel between sites. Similar concerns have been raised by UK studies, in which also the provision of specialist equipment to multiple sites was seen as a challenge [16, 35, 47]. As there are less referrals to the hospital in the intervention, and patients referred might be more appropriately investigated already in primary care, less contacts, time and resources are needed in the hospital compared to the standard care pathway. If this is enough to compensate for the resource moved from hospital to primary care, cannot be directly said based on this study. Also, the opportunity cost of the time of the dermatologist is not known. This is a common challenge preventing effective prioritization of healthcare. The cost-effectiveness of the intervention care pathway is reported elsewhere [Lovén et al. 2024. The article “The integration of dermatology experts into primary care to assess and treat patients with skin lesions is cost-effective: a quasi-experimental study” accepted for publication in JEADV]. Equipment supply was not considered to be problematic in our study.

Aside from the intervention itself and the other confounders for which we adjusted, other factors may have affected our findings. We accept that the addition of a dermatologist may have a broader impact at a health centre than a simple augmentation in specialist expertise. In our intervention the dermatologist was a consultant from outside the organisation and therefore could have increased the overall available resource by performing the assessments and treatment of skin conditions that had previously been covered by the GPs as part of their normal work. In the intervention centres, the dermatologist represented 1.2% of the total doctor resource (0.15 full-time equivalent). Due to substantial variation in general practitioner resources across different days and months at the health centres (over ± 20% of the total resource), it is difficult to evaluate to what degree, if any, the integration of the dermatologist added the total available resource, thereby influencing diagnosis and treatment timing. Since the dermatologists were not present at the clinics every day, the intervention itself may even have caused short delays because it is possible that patients could have visited a GP sooner rather than waiting for the next day the dermatologist was available. We compared overall access to resource among our study centres using the ‘third next available appointment’ (TNAA) measure [48]. For doctor appointments, the median TNAA was 3.5 days in Keminmaa (intervention) and 7.0 days in both Tornio (intervention) and Kemi (control). For nurse appointments, the median TNAA was 2.0 days in Keminmaa (intervention), 1.5 days in Tornio (intervention) and 1.0 day in Kemi (control). These findings indicate no marked difference between the sites in terms of general access to healthcare resource.

### Strengths and limitations

This practice-based research documented the real-world effects of integrating dermatologist within an operational setting. A randomized controlled trial would probably have presented a higher internal validity, while the results of a practice-based study may possess greater external validity—immediately suitable for ‘real-world’ implementation [49, 50].

The main limitation was the lack of professionals' perspectives on the old care path after the follow-up period. The professional satisfaction questionnaires were not returned by the physicians at the control centre as required by the study design, before modifications to the care path. Since the control centre subsequently took up the integrated model of treatment as everyday practice, the investigators offered the staff at that centre the opportunity to give feedback on the same questionnaire completed by professionals at the intervention centres during the study. Since this constituted a breach of study design, the ‘post-intervention’ responses in the professionals’ control group must be interpreted with caution. The adoption of the integrated care path led to the formation of another pre-post intervention study group in professionals’ questionnaires (Fig. 2).

Although our population was large enough to test for statistical significance, it could be said that a larger population, perhaps spread among more centres, may have yielded more reliable and generalizable results. One limitation is the missing data from patients who refused to participate. However, based on medical staff reports and schedules, we know that only a few declined attendance, making it improbable that this significantly impacted the results.

A potential limitation that might affect the implications for practice in some countries might be the scarcity of dermatologists [51, 52].

The strength is that the population in our study is presumed to represent the adult primary care demographic; it includes all the primary care centres on the area, where minimal private alternatives exist, and encompasses the majority of patients presenting with skin changes within a specified period. The timelines and delays were collected from the records to the true initiation of treatment. Validated, standardized measures were included in the satisfaction survey, especially PEI Q2, which has been found to well measure the patient perceived benefit [27]. This study is among the first to investigate the effects of the partial replacement of a GP with a specialist in a primary care setting for the assessment and treatment of a specific patient group. A carefully planned replacement has significant potential for saving time and resources. 

### Implications for future research

For further research, the specific indications for direct dermatologist appointments in primary care, along with an optimized daily schedule could be investigated. A similar replacement model, with clear indications for direct appointments and effective daily organization, could be applied to other specialties that do not require extensive equipment investments.

## Conclusions

Our findings suggest that integrating dermatological expertise into primary care, by replacing a GP for certain tasks, could streamline the management of patients with skin disorders. Integration may reduce diagnostic and treatment delays, enhance patient and professional experiences, and decrease the number of visits while simultaneously increasing the proportion of patients treated in primary care. Regardless of the healthcare funding structure, the presented model and results can be broadly applied globally.

## Supplementary Information

Additional file 1. Patient experience questionnaire.

Additional file 2. Professional experience start questionnaire.

Additional file 3. Professional experience follow-up questionnaire.

Additional file 4: Tables S1-S5. include regression model results of the outcomes. Tables S6-S7. include pre- and post-intervention characteristics of the professionals, comparison between groups.

## Data Availability

Upon reasonable request the corresponding author will share the dataset considering experience questionnaires and the description to enable the replication of data extraction from health care providers’databases. The ethical permission or patient consent does not allow to share the full data.

## Abbreviations

AIC: Akaike information criterion
GP: General practitioner
NPS: Net Promoter Score
PAD: Pathological-anatomic diagnosis
PEI: Patient Enablement Instrument
Q1: Quartile 1
Q2: Question 2
Q3: Quartile 3
TNAA: Third next available appointment
