# Supplementary material for: Integrating dermatologists in primary care: impact on delays, patient and professional experiences
Source: BMC Health Serv Res. 2024 Nov 20;24:1441. doi: 10.1186/s12913-024-11923-y (PMC11577956; doi:10.1186/s12913-024-11923-y)
Supplement: Supplementary file 1 — Additional file 1. Patient experience questionnaire. [file 12913_2024_11923_MOESM1_ESM.docx]

**Additional file 1**

Name: Additional file 1 NPS and PEI

Format: word-document (docx)

Title: Additional file 1

Description: Patient experience questionnaire

Patient Enablement Instrument (PEI), Question 2

As a result of your visit to the doctor today, do you feel you are able to cope with your illness...

Much better…….………………..

Better……………………………..

Same or less…………………….

NPS Question

Would you recommend Mehiläinen Länsi-Pohja's services? Please answer with a number from 0-10 (10=very likely). After the number, you can write open feedback. Thank you!
