# Supplementary material for: Integrating dermatologists in primary care: impact on delays, patient and professional experiences
Source: BMC Health Serv Res. 2024 Nov 20;24:1441. doi: 10.1186/s12913-024-11923-y (PMC11577956; doi:10.1186/s12913-024-11923-y)
Supplement: Supplementary file 2 — Additional file 2. Professional experience start questionnaire. [file 12913_2024_11923_MOESM2_ESM.docx]

**Additional file 2**

Name: Additional file 2 Professional_Experience_start

Format: word-document (docx)

Title: Additional file 2

Description: Professional experience start questionnaire

**Satisfaction with the Current Care Model for Dermatology Patients**

1. Date __**/__** 202_
2. Health Center, please circle the correct one:

1. Tornio 2. Keminmaa 3. Kemi

1. Respondent's Professional Group, please circle the correct one.

1. Specialist Doctor 2. Licensed Physician 3. Medical Student 4. Nurse / Public Health Nurse 5. Other, please specify:

1. How long have you worked at the health center? Please circle the correct option.
   - 1. Less than 3 months 2. 3-9 months 3. More than 9 months

5. Have you treated or referred dermatology patients for further treatment?

1. Yes 2. No

1. How satisfied are you on a scale of 1-5 with the new care model for dermatology patients - where the dermatologist visits the health center?

1. Very satisfied 2. Satisfied 3. Neither satisfied nor dissatisfied 4. Dissatisfied 5. Very dissatisfied

1. Would you recommend the new care pathway for dermatology patients, where the dermatologist makes the initial assessment of the patient's skin changes, to a colleague? Answer with a number 0-10 (10=very likely, 0=not likely). After the number, you can write free feedback.

10 9 8 7 6 5 4 3 2 1 0

Would you please answer the following open-ended questions:

1. Which aspect of the current care pathway for dermatology patients are you dissatisfied with?
2. Which aspect of the current care pathway for dermatology patients are you satisfied with?
3. How would you improve the care pathway for dermatology patients in the health center, considering collaboration with the hospital?

Thank you for your responses!
