# Supplementary material for: Integrating dermatologists in primary care: impact on delays, patient and professional experiences
Source: BMC Health Serv Res. 2024 Nov 20;24:1441. doi: 10.1186/s12913-024-11923-y (PMC11577956; doi:10.1186/s12913-024-11923-y)
Supplement: Supplementary file 3 — Additional file 3. Professional experience follow-up questionnaire. [file 12913_2024_11923_MOESM3_ESM.docx]

**Additional file 3**

Name: Additional file 3 Professional_Experience_follow-up

Format: word-document (docx)

Title: Additional file 3

Description: Professional experience follow-up questionnaire

**Follow-up Survey for Outpatient Staff** **Satisfaction with the New Care Pathway for Dermatology Patients, where a Dermatologist Visits the Health Center**

1. Date __**/__** 202_
2. Health Center, please circle the correct one:

1. Tornio 2. Keminmaa 3. Kemi

1. Respondent's Professional Group, please circle the correct one.

1. Specialist Doctor 2. Licensed Physician 3. Medical Student 4. Nurse / Public Health Nurse 5. Other, please specify:

1. How long have you worked at the health center? Please circle the correct option.
   - 1. Less than 3 months 2. 3-9 months 3. More than 9 months

5. a. Have you treated or referred dermatology patients during the traditional care pathway (before the dermatologist started visiting the health center)?

1. Yes 2. No

1. b. Have you treated or referred dermatology patients during the new care pathway (after February 2021) with the dermatologist working at the health center?

1. Yes 2. No

1. How satisfied are you on a scale of 1-5 with the new care model for dermatology patients - where the dermatologist visits the health center?

1. Very satisfied 2. Satisfied 3. Neither satisfied nor dissatisfied 4. Dissatisfied 5. Very dissatisfied

1. Would you recommend the new care pathway for dermatology patients, where the dermatologist makes the initial assessment of the patient's skin changes, to a colleague? Answer with a number 0-10 (10=very likely, 0=not likely). After the number, you can write free feedback.

10 9 8 7 6 5 4 3 2 1 0

How has the new care pathway affected the treatment of dermatology patients? Please circle the appropriate options. Answer in more detail if you wish.

1. The new care pathway has been more beneficial for patients than the previous model. How?
   - - 1. Yes 2. No
2. The new care model has been worse for patients than the previous model. How?
   - - 1. Yes 2. No
3. The care model has reduced my workload in patient care, how?
   - - 1. Yes 2. No
4. The care model has increased my workload in patient care, how?
   - - 1. Yes 2. No
5. Referring dermatology patients for treatment has become
   - - 1. Easier 2. More difficult 3. No difference compared to before
6. I find the new care model valuable and hope it continues.
   - - 1. Yes 2. No
7. Have you felt that your competence in treating dermatology patients has improved with the new care model?
   - 1. A lot 2. Somewhat 3. Not at all
8. Which of the following have improved your competence in treating dermatology patients?
   - 1. Specialist's lecture-based training at the health center
     2. 2 Specialist's consultation availability at the health center
     3. 3 Specialist's hands-on teaching
     4. Consultation with a hospital specialist
     5. Working as part of the dermatology patient care pathway (e.g., assisting the dermatologist)
     6. Discussions with colleagues or other professional groups
     7. Self-study
     8. External training
     9. Other, please specify:

Would you please answer the following open-ended questions:

1. Which aspect of the new care model for dermatology patients are you dissatisfied with?
2. Which aspect of the new care model for dermatology patients are you satisfied with?
3. How would you further develop the care model for dermatology patients in your health center, including collaboration with the Länsi-Pohja Central Hospital? What would make the dermatologist's days more beneficial for your learning?

Thank you for your responses!
