# Supplementary material for: Integrating dermatologists in primary care: impact on delays, patient and professional experiences
Source: BMC Health Serv Res. 2024 Nov 20;24:1441. doi: 10.1186/s12913-024-11923-y (PMC11577956; doi:10.1186/s12913-024-11923-y)
Supplement: Supplementary file 4 — Additional file 4: Tables S1-S5. include regression model results of the outcomes. Tables S6-S7. include pre- and post-intervention characteristics of the professionals, comparison between groups. [file 12913_2024_11923_MOESM4_ESM.docx]

**Additional file 4**

Name: Additional file 4

Format: word-document (docx)

Title: Additional file 4

Description: Tables S1 - S5 include regression model results of the outcomes. Tables S6 - S7 include pre- and post-intervention characteristics of the professionals, comparison between groups.

**Table S1.** Linear regression for the continuous process outcomes, adjuster for gender, age, smoking, multimorbidity and previous skin diseases. Effect of the intervention bolded.

| **Variables** | **Term** | **Model** | **Estimate** | **Standard error** | **Statistic** | **p value** | **r squared** | **Aic** |
| --- | --- | --- | --- | --- | --- | --- | --- | --- |
| Number of diagnosis | crude | Intercept | 2,8 | 0,1 | 40,2 | 0,00 | 0,5 | 943 |
| **Number of diagnosis** | **crude** | **Group: Control** | **-1,8** | **0,1** | **-17,5** | **0,00** | **0,5** | **943** |
| Number of diagnosis | adjusted | Intercept | 2,5 | 0,2 | 10,2 | 0,00 | 0,5 | 936 |
| **Number of diagnosis** | **adjusted** | **Group: Control** | **-1,8** | **0,1** | **-17,2** | **0,00** | **0,5** | **936** |
| Number of diagnosis | adjusted | Age | 0,0 | 0,0 | 2,8 | 0,01 | 0,5 | 936 |
| Number of diagnosis | adjusted | Previous Skin diseases: Yes | 0,0 | 0,1 | 0,0 | 0,98 | 0,5 | 936 |
| Number of diagnosis | adjusted | Multimorbidity: Yes | -0,1 | 0,1 | -0,9 | 0,37 | 0,5 | 936 |
| Number of diagnosis | adjusted | Non-smoking | -0,1 | 0,2 | -0,3 | 0,77 | 0,5 | 936 |
| Number of diagnosis | adjusted | Ex-smoker | 0,0 | 0,2 | -0,1 | 0,90 | 0,5 | 936 |
| Number of diagnosis | adjusted | Smoking | -0,2 | 0,2 | -0,9 | 0,34 | 0,5 | 936 |
| Number of diagnosis | adjusted | Sex: Female | -0,2 | 0,1 | -1,4 | 0,16 | 0,5 | 936 |
| Contacts until diagnosis | crude | Intercept | 3,4 | 0,2 | 20,7 | 0,00 | 0,1 | 1610 |
| **Contacts until diagnosis** | **crude** | **Group: Control** | **1,1** | **0,2** | **4,5** | **0,00** | **0,1** | **1610** |
| Contacts until diagnosis | adjusted | Intercept | 3,3 | 0,6 | 5,8 | 0,00 | 0,1 | 1603 |
| **Contacts until diagnosis** | **adjusted** | **Group: Control** | **1,1** | **0,2** | **4,6** | **0,00** | **0,1** | **1603** |
| Contacts until diagnosis | adjusted | Age | 0,0 | 0,0 | 0,2 | 0,88 | 0,1 | 1603 |
| Contacts until diagnosis | adjusted | Previous Skin diseases: Yes | 0,5 | 0,3 | 2,1 | 0,04 | 0,1 | 1603 |
| Contacts until diagnosis | adjusted | Multimorbidity: Yes | -0,3 | 0,3 | -0,9 | 0,36 | 0,1 | 1603 |
| Contacts until diagnosis | adjusted | Non-smoking | 0,1 | 0,4 | 0,2 | 0,85 | 0,1 | 1603 |
| Contacts until diagnosis | adjusted | Ex-smoker | 0,1 | 0,4 | 0,2 | 0,81 | 0,1 | 1603 |
| Contacts until diagnosis | adjusted | Smoking | 0,4 | 0,5 | 0,9 | 0,37 | 0,1 | 1603 |
| Contacts until diagnosis | adjusted | Sex: Female | -0,3 | 0,3 | -1,2 | 0,23 | 0,1 | 1603 |
| Contacts | crude | Intercept | 4,1 | 0,2 | 16,7 | 0,00 | 0,1 | 1898 |
| **Contacts** | **crude** | **Group: Control** | **1,9** | **0,3** | **5,4** | **0,00** | **0,1** | **1898** |
| Contacts | adjusted | Intercept | 2,5 | 0,8 | 3,0 | 0,00 | 0,1 | 1879 |
| **Contacts** | **adjusted** | **Group: Control** | **2,0** | **0,4** | **5,7** | **0,00** | **0,1** | **1879** |
| Contacts | adjusted | Age | 0,0 | 0,0 | 2,4 | 0,02 | 0,1 | 1879 |
| Contacts | adjusted | Previous Skin diseases: Yes | 0,9 | 0,4 | 2,4 | 0,02 | 0,1 | 1879 |
| Contacts | adjusted | Multimorbidity: Yes | -0,8 | 0,4 | -2,0 | 0,05 | 0,1 | 1879 |
| Contacts | adjusted | Non-smoking | 0,5 | 0,6 | 1,0 | 0,34 | 0,1 | 1879 |
| Contacts | adjusted | Ex-smoker | 0,0 | 0,7 | 0,0 | 0,97 | 0,1 | 1879 |
| Contacts | adjusted | Smoking | 0,7 | 0,7 | 1,0 | 0,33 | 0,1 | 1879 |
| Contacts | adjusted | Sex: Female | -0,6 | 0,4 | -1,7 | 0,10 | 0,1 | 1879 |
| Delay to confirmed diagnosis | crude | Intercept | 35,1 | 2,6 | 13,5 | 0,00 | 0,1 | 2746 |
| **Delay to confirmed diagnosis** | **crude** | **Group: Control** | **30,1** | **4,6** | **6,6** | **0,00** | **0,1** | **2746** |
| Delay to confirmed diagnosis | adjusted | (Intercept) | 43,5 | 10,4 | 4,2 | 0,00 | 0,2 | 2733 |
| **Delay to confirmed diagnosis** | **adjusted** | **Group: Control** | **30,4** | **4,7** | **6,5** | **0,00** | **0,2** | **2733** |
| Delay to confirmed diagnosis | adjusted | Age | -0,2 | 0,1 | -1,6 | 0,11 | 0,2 | 2733 |
| Delay to confirmed diagnosis | adjusted | Previous Skin diseases: Yes | 4,0 | 4,6 | 0,9 | 0,38 | 0,2 | 2733 |
| Delay to confirmed diagnosis | adjusted | Multimorbidity: Yes | -2,8 | 5,1 | -0,6 | 0,58 | 0,2 | 2733 |
| Delay to confirmed diagnosis | adjusted | Non-smoking | -0,7 | 6,8 | -0,1 | 0,92 | 0,2 | 2733 |
| Delay to confirmed diagnosis | adjusted | Ex-smoker | 6,8 | 8,0 | 0,9 | 0,40 | 0,2 | 2733 |
| Delay to confirmed diagnosis | adjusted | Smoking | -6,1 | 8,8 | -0,7 | 0,49 | 0,2 | 2733 |
| Delay to confirmed diagnosis | adjusted | Sex: Female | 7,2 | 4,8 | 1,5 | 0,14 | 0,2 | 2733 |
| Delay to first diagnosis | crude | Intercept | 28,1 | 2,3 | 12,5 | 0,00 | 0,0 | 3450 |
| **Delay to first diagnosis** | **crude** | **Group: Control** | **8,0** | **3,2** | **2,5** | **0,01** | **0,0** | **3450** |
| Delay to first diagnosis | adjusted | (Intercept) | 31,2 | 7,9 | 3,9 | 0,00 | 0,0 | 3431 |
| **Delay to first diagnosis** | **adjusted** | **Group: Control** | **9,0** | **3,4** | **2,7** | **0,01** | **0,0** | **3431** |
| Delay to first diagnosis | adjusted | Age | 0,0 | 0,1 | 0,3 | 0,74 | 0,0 | 3431 |
| Delay to first diagnosis | adjusted | Previous Skin diseases: Yes | 3,8 | 3,5 | 1,1 | 0,29 | 0,0 | 3431 |
| Delay to first diagnosis | adjusted | Multimorbidity: Yes | -2,5 | 3,8 | -0,7 | 0,51 | 0,0 | 3431 |
| Delay to first diagnosis | adjusted | Non-smoking | -8,6 | 5,4 | -1,6 | 0,11 | 0,0 | 3431 |
| Delay to first diagnosis | adjusted | Ex-smoker | -7,5 | 6,1 | -1,2 | 0,22 | 0,0 | 3431 |
| Delay to first diagnosis | adjusted | Smoking | -11,1 | 6,9 | -1,6 | 0,11 | 0,0 | 3431 |
| Delay to first diagnosis | adjusted | Sex: Female | 2,8 | 3,6 | 0,8 | 0,44 | 0,0 | 3431 |
| Delay from main visit to start to treat | crude | (Intercept) | 4,7 | 3,4 | 1,4 | 0,17 | 0,1 | 1543 |
| **Delay from main visit to start to treat** | **crude** | **Group: Control** | **19,6** | **5,5** | **3,5** | **0,00** | **0,1** | **1543** |
| Delay from main visit to start to treat | adjusted | Intercept | -30,4 | 14,6 | -2,1 | 0,04 | 0,1 | 1536 |
| **Delay from main visit to start to treat** | **adjusted** | **Group: Control** | **21,1** | **5,7** | **3,7** | **0,00** | **0,1** | **1536** |
| Delay from main visit to start to treat | adjusted | Age | 0,4 | 0,2 | 2,6 | 0,01 | 0,1 | 1536 |
| Delay from main visit to start to treat | adjusted | Previous Skin diseases: Yes | 0,3 | 5,7 | 0,1 | 0,96 | 0,1 | 1536 |
| Delay from main visit to start to treat | adjusted | Multimorbidity: Yes | -5,7 | 6,5 | -0,9 | 0,38 | 0,1 | 1536 |
| Delay from main visit to start to treat | adjusted | Non-smoking | 11,6 | 10,7 | 1,1 | 0,28 | 0,1 | 1536 |
| Delay from main visit to start to treat | adjusted | Ex-smoker | 3,8 | 12,0 | 0,3 | 0,75 | 0,1 | 1536 |
| Delay from main visit to start to treat | adjusted | Smoking | 2,0 | 12,7 | 0,2 | 0,88 | 0,1 | 1536 |
| Delay from main visit to start to treat | adjusted | Sex: Female | 2,1 | 5,8 | 0,4 | 0,72 | 0,1 | 1536 |
| Delay from first contact to start to treat* | crude | (Intercept) | 31,9 | 4,6 | 6,9 | 0,00 | 0,1 | 1603 |
| **Delay from first contact to start to treat*** | **crude** | **Group: Control** | **30,7** | **7,5** | **4,1** | **0,00** | **0,1** | **1603** |
| Delay from first contact to start to treat* | adjusted | Intercept | -25,3 | 19,2 | -1,3 | 0,19 | 0,2 | 1587 |
| **Delay from first contact to start to treat*** | **adjusted** | **Group: Control** | **31,9** | **7,5** | **4,2** | **0,00** | **0,2** | **1587** |
| Delay from first contact to start to treat* | adjusted | Age | 0,9 | 0,2 | 3,9 | 0,00 | 0,2 | 1587 |
| Delay from first contact to start to treat* | adjusted | Previous Skin diseases: Yes | -0,9 | 7,5 | -0,1 | 0,90 | 0,2 | 1587 |
| Delay from first contact to start to treat* | adjusted | Multimorbidity: Yes | -17,3 | 8,5 | -2,0 | 0,04 | 0,2 | 1587 |
| Delay from first contact to start to treat* | adjusted | Non-smoking | 11,9 | 13,9 | 0,9 | 0,39 | 0,2 | 1587 |
| Delay from first contact to start to treat* | adjusted | Ex-smoker | 4,4 | 15,7 | 0,3 | 0,78 | 0,2 | 1587 |
| Delay from first contact to start to treat* | adjusted | Smoking | 0,3 | 16,9 | 0,0 | 0,99 | 0,2 | 1587 |
| Delay from first contact to start to treat* | adjusted | Sex: Female | 2,2 | 7,7 | 0,3 | 0,77 | 0,2 | 1587 |
| Delay from first contact to main visit | crude | (Intercept) | 30,6 | 2,5 | 12,2 | 0,00 | 0,0 | 3586 |
| **Delay from first contact to main visit** | **crude** | **Group: Control** | **14,6** | **3,6** | **4,1** | **0,00** | **0,0** | **3586** |
| Delay from first contact to main visit | adjusted | Intercept | 28,6 | 8,8 | 3,3 | 0,00 | 0,1 | 3567 |
| **Delay from first contact to main visit** | **adjusted** | **Group: Control** | **15,2** | **3,7** | **4,1** | **0,00** | **0,1** | **3567** |
| Delay from first contact to main visit | adjusted | Age | 0,2 | 0,1 | 1,3 | 0,19 | 0,1 | 3567 |
| Delay from first contact to main visit | adjusted | Previous Skin diseases: Yes | 2,8 | 3,9 | 0,7 | 0,48 | 0,1 | 3567 |
| Delay from first contact to main visit | adjusted | Multimorbidity: Yes | -8,3 | 4,2 | -2,0 | 0,05 | 0,1 | 3567 |
| Delay from first contact to main visit | adjusted | Non-smoking | -3,6 | 6,0 | -0,6 | 0,55 | 0,1 | 3567 |
| Delay from first contact to main visit | adjusted | Ex-smoker | -4,9 | 6,8 | -0,7 | 0,48 | 0,1 | 3567 |
| Delay from first contact to main visit | adjusted | Smoking | -4,4 | 7,7 | -0,6 | 0,57 | 0,1 | 3567 |
| Delay from first contact to main visit | adjusted | Sex: Female | -1,3 | 4,0 | -0,3 | 0,74 | 0,1 | 3567 |

*Three patients missing of the variable “Delay from first contact to start to treat”, otherwise no missing data.**Table S2.** Linear regression for the delays, adjuster for gender, age, smoking, multimorbidity, previous skin diseases and delay between first contact to doctor appointment. Effect of the intervention bolded.

| **Variables** | **Term** | **Model** | **Estimate** | **Standard error** | **Statistic** | **p value** | **r2** | **Aic** |
| --- | --- | --- | --- | --- | --- | --- | --- | --- |
| Number of diagnosis | Intercept | crude | 2,8 | 0,1 | 40,2 | 0,00 | 0,5 | 943 |
| **Number of diagnosis** | **Group: Control** | **crude** | **-1,8** | **0,1** | **-17,5** | **0,00** | **0,5** | **943** |
| Number of diagnosis | Intercept | adjusted | 2,5 | 0,2 | 10,1 | 0,00 | 0,5 | 938 |
| **Number of diagnosis** | **Group: Control** | **adjusted** | **-1,8** | **0,1** | **-16,6** | **0,00** | **0,5** | **938** |
| Number of diagnosis | Age | adjusted | 0,0 | 0,0 | 2,8 | 0,01 | 0,5 | 938 |
| Number of diagnosis | Previous Skin diseases: Yes | adjusted | 0,0 | 0,1 | 0,0 | 1,00 | 0,5 | 938 |
| Number of diagnosis | Multimorbidity: Yes | adjusted | -0,1 | 0,1 | -0,9 | 0,35 | 0,5 | 938 |
| Number of diagnosis | Non-smoking | adjusted | -0,1 | 0,2 | -0,3 | 0,75 | 0,5 | 938 |
| Number of diagnosis | Ex-smoker | adjusted | 0,0 | 0,2 | -0,2 | 0,88 | 0,5 | 938 |
| Number of diagnosis | Smoking | adjusted | -0,2 | 0,2 | -1,0 | 0,33 | 0,5 | 938 |
| Number of diagnosis | Sex: Female | adjusted | -0,2 | 0,1 | -1,4 | 0,16 | 0,5 | 938 |
| Number of diagnosis | Delay from first contact to main visit | adjusted | 0,0 | 0,0 | -0,5 | 0,61 | 0,5 | 938 |
| Contacts until diagnosis | Intercept | crude | 3,4 | 0,2 | 20,7 | 0,00 | 0,1 | 1610 |
| **Contacts until diagnosis** | **Group: Control** | **crude** | **1,1** | **0,2** | **4,5** | **0,00** | **0,1** | **1610** |
| Contacts until diagnosis | Intercept | adjusted | 2,7 | 0,5 | 5,0 | 0,00 | 0,2 | 1567 |
| **Contacts until diagnosis** | **Group: Control** | **adjusted** | **0,8** | **0,2** | **3,4** | **0,00** | **0,2** | **1567** |
| Contacts until diagnosis | Age | adjusted | 0,0 | 0,0 | -0,3 | 0,78 | 0,2 | 1567 |
| Contacts until diagnosis | Previous Skin diseases: Yes | adjusted | 0,5 | 0,2 | 1,9 | 0,05 | 0,2 | 1567 |
| Contacts until diagnosis | Multimorbidity: Yes | adjusted | -0,1 | 0,3 | -0,3 | 0,76 | 0,2 | 1567 |
| Contacts until diagnosis | Non-smoking | adjusted | 0,1 | 0,4 | 0,4 | 0,69 | 0,2 | 1567 |
| Contacts until diagnosis | Ex-smoker | adjusted | 0,2 | 0,4 | 0,5 | 0,62 | 0,2 | 1567 |
| Contacts until diagnosis | Smoking | adjusted | 0,5 | 0,5 | 1,1 | 0,26 | 0,2 | 1567 |
| Contacts until diagnosis | Sex: Female | adjusted | -0,3 | 0,2 | -1,2 | 0,24 | 0,2 | 1567 |
| Contacts until diagnosis | Delay from first contact to main visit | adjusted | 0,0 | 0,0 | 6,3 | 0,00 | 0,2 | 1567 |
| Contacts | Intercept | crude | 4,1 | 0,2 | 16,7 | 0,00 | 0,1 | 1898 |
| **Contacts** | **Group: Control** | **crude** | **1,9** | **0,3** | **5,4** | **0,00** | **0,1** | **1898** |
| Contacts | Intercept | adjusted | 1,8 | 0,8 | 2,2 | 0,03 | 0,2 | 1858 |
| **Contacts** | **Group: Control** | **adjusted** | **1,6** | **0,4** | **4,7** | **0,00** | **0,2** | **1858** |
| Contacts | Age | adjusted | 0,0 | 0,0 | 2,1 | 0,03 | 0,2 | 1858 |
| Contacts | Previous Skin diseases: Yes | adjusted | 0,8 | 0,4 | 2,2 | 0,03 | 0,2 | 1858 |
| Contacts | Multimorbidity: Yes | adjusted | -0,6 | 0,4 | -1,5 | 0,13 | 0,2 | 1858 |
| Contacts | Non-smoking | adjusted | 0,6 | 0,6 | 1,1 | 0,25 | 0,2 | 1858 |
| Contacts | Ex-smoker | adjusted | 0,1 | 0,6 | 0,2 | 0,83 | 0,2 | 1858 |
| Contacts | Smoking | adjusted | 0,8 | 0,7 | 1,2 | 0,25 | 0,2 | 1858 |
| Contacts | Sex: Female | adjusted | -0,6 | 0,4 | -1,6 | 0,11 | 0,2 | 1858 |
| Contacts | Delay from first contact to main visit | adjusted | 0,0 | 0,0 | 4,8 | 0,00 | 0,2 | 1858 |
| Delay to confirmed diagnosis | Intercept | crude | 35,1 | 2,6 | 13,5 | 0,00 | 0,1 | 2746 |
| **Delay to confirmed diagnosis** | **Group: Control** | **crude** | **30,1** | **4,6** | **6,6** | **0,00** | **0,1** | **2746** |
| Delay to confirmed diagnosis | Intercept | adjusted | 12,7 | 7,2 | 1,8 | 0,08 | 0,6 | 2515 |
| **Delay to confirmed diagnosis** | **Group: Control** | **adjusted** | **17,8** | **3,2** | **5,6** | **0,00** | **0,6** | **2515** |
| Delay to confirmed diagnosis | Age | adjusted | -0,2 | 0,1 | -1,8 | 0,08 | 0,6 | 2515 |
| Delay to confirmed diagnosis | Previous Skin diseases: Yes | adjusted | 2,5 | 3,1 | 0,8 | 0,41 | 0,6 | 2515 |
| Delay to confirmed diagnosis | Multimorbidity: Yes | adjusted | 1,7 | 3,4 | 0,5 | 0,63 | 0,6 | 2515 |
| Delay to confirmed diagnosis | Non-smoking | adjusted | 3,9 | 4,6 | 0,8 | 0,40 | 0,6 | 2515 |
| Delay to confirmed diagnosis | Ex-smoker | adjusted | 8,2 | 5,4 | 1,5 | 0,13 | 0,6 | 2515 |
| Delay to confirmed diagnosis | Smoking | adjusted | -1,0 | 5,9 | -0,2 | 0,87 | 0,6 | 2515 |
| Delay to confirmed diagnosis | Sex: Female | adjusted | 2,4 | 3,2 | 0,7 | 0,45 | 0,6 | 2515 |
| Delay to confirmed diagnosis | Delay from first contact to main visit | adjusted | 0,9 | 0,0 | 18,0 | 0,00 | 0,6 | 2515 |
| Delay to first diagnosis | Intercept | crude | 28,1 | 2,3 | 12,5 | 0,00 | 0,0 | 3450 |
| **Delay to first diagnosis** | **Group: Control** | **crude** | **8,0** | **3,2** | **2,5** | **0,01** | **0,0** | **3450** |
| Delay to first diagnosis | Intercept | adjusted | 10,4 | 5,1 | 2,0 | 0,04 | 0,6 | 3113 |
| **Delay to first diagnosis** | **Group: Control** | **adjusted** | **-2,2** | **2,2** | **-1,0** | **0,31** | **0,6** | **3113** |
| Delay to first diagnosis | Age | adjusted | -0,1 | 0,1 | -0,8 | 0,43 | 0,6 | 3113 |
| Delay to first diagnosis | Previous Skin diseases: Yes | adjusted | 2,5 | 2,2 | 1,1 | 0,26 | 0,6 | 3113 |
| Delay to first diagnosis | Multimorbidity: Yes | adjusted | 3,7 | 2,4 | 1,5 | 0,13 | 0,6 | 3113 |
| Delay to first diagnosis | Non-smoking | adjusted | -5,7 | 3,4 | -1,6 | 0,10 | 0,6 | 3113 |
| Delay to first diagnosis | Ex-smoker | adjusted | -4,5 | 3,9 | -1,1 | 0,25 | 0,6 | 3113 |
| Delay to first diagnosis | Smoking | adjusted | -8,0 | 4,4 | -1,8 | 0,07 | 0,6 | 3113 |
| Delay to first diagnosis | Sex: Female | adjusted | 2,8 | 2,3 | 1,2 | 0,22 | 0,6 | 3113 |
| Delay to first diagnosis | Delay from first contact to main visit | adjusted | 0,7 | 0,0 | 22,5 | 0,00 | 0,6 | 3113 |
| Delay from main visit to start to treat | Intercept | crude | 4,7 | 3,4 | 1,4 | 0,17 | 0,1 | 1543 |
| **Delay from main visit to start to treat** | **Group: Control** | **crude** | **19,6** | **5,5** | **3,5** | **0,00** | **0,1** | **1543** |
| **Delay from main visit to start to treat** | **Intercept** | **adjusted** | **-30,7** | **14,7** | **-2,1** | **0,04** | **0,1** | **1538** |
| **Delay from main visit to start to treat** | **Group: Control** | **adjusted** | **20,9** | **5,8** | **3,6** | **0,00** | **0,1** | **1538** |
| Delay from main visit to start to treat | Age | adjusted | 0,4 | 0,2 | 2,5 | 0,01 | 0,1 | 1538 |
| Delay from main visit to start to treat | Previous Skin diseases: Yes | adjusted | 0,3 | 5,7 | 0,1 | 0,96 | 0,1 | 1538 |
| Delay from main visit to start to treat | Multimorbidity: Yes | adjusted | -5,5 | 6,6 | -0,8 | 0,40 | 0,1 | 1538 |
| Delay from main visit to start to treat | Non-smoking | adjusted | 11,6 | 10,7 | 1,1 | 0,28 | 0,1 | 1538 |
| Delay from main visit to start to treat | Ex-smoker | adjusted | 3,8 | 12,0 | 0,3 | 0,75 | 0,1 | 1538 |
| Delay from main visit to start to treat | Smoking | adjusted | 2,1 | 12,8 | 0,2 | 0,87 | 0,1 | 1538 |
| Delay from main visit to start to treat | Sex: Female | adjusted | 2,2 | 5,9 | 0,4 | 0,71 | 0,1 | 1538 |
| Delay from main visit to start to treat* | Delay from first contact to main visit | adjusted | 0,0 | 0,1 | 0,2 | 0,84 | 0,1 | 1538 |
| Delay from first contact to start to treat* | Intercept | crude | 31,9 | 4,6 | 6,9 | 0,00 | 0,1 | 1603 |
| **Delay from first contact to start to treat*** | **Group: Control** | **crude** | **30,7** | **7,5** | **4,1** | **0,00** | **0,1** | **1603** |
| Delay from first contact to start to treat* | Intercept | adjusted | -39,2 | 14,7 | -2,7 | 0,01 | 0,5 | 1507 |
| **Delay from first contact to start to treat*** | **Group: Control** | **adjusted** | **20,9** | **5,8** | **3,6** | **0,00** | **0,5** | **1507** |
| Delay from first contact to start to treat* | Age | adjusted | 0,6 | 0,2 | 3,4 | 0,00 | 0,5 | 1507 |
| Delay from first contact to start to treat* | Previous Skin diseases: Yes | adjusted | -0,2 | 5,7 | 0,0 | 0,98 | 0,5 | 1507 |
| Delay from first contact to start to treat* | Multimorbidity: Yes | adjusted | -8,0 | 6,6 | -1,2 | 0,22 | 0,5 | 1507 |
| Delay from first contact to start to treat* | Non-smoking | adjusted | 8,7 | 10,6 | 0,8 | 0,42 | 0,5 | 1507 |
| Delay from first contact to start to treat* | Ex-smoker | adjusted | 6,6 | 12,0 | 0,5 | 0,58 | 0,5 | 1507 |
| Delay from first contact to start to treat* | Smoking | adjusted | 3,7 | 12,9 | 0,3 | 0,78 | 0,5 | 1507 |
| Delay from first contact to start to treat* | Sex: Female | adjusted | 5,5 | 5,9 | 0,9 | 0,35 | 0,5 | 1507 |
| Delay from first contact to start to treat* | Delay from first contact to main visit | adjusted | 0,9 | 0,1 | 10,1 | 0,00 | 0,5 | 1507 |

*Three patients missing of the variable “Delay from first contact to start to treat”, otherwise no missing data.

**Table S3.** The result of logistic regression analysis for treatment started, crude and adjusted values. No missing data.

| Response variables | Treatment started in Primary care | Treatment started at all |
| --- | --- | --- |
| Crude OR | 0,37 | 0,46 |
| Crude CI lower | 0,24 | 0,30 |
| Crude CI upper | 0,58 | 0,71 |
| Crude p | 0,000 | 0,000 |
| Adjusted OR | 0,36 | 0,46 |
| Adjusted CI lower | 0,23 | 0,29 |
| Adjusted CI upper | 0,57 | 0,72 |
| Adjusted p | 0,000 | 0,001 |
| Crude aic | 466 | 486 |
| Adjusted aic | 459 | 472 |
| Missing data | 0 | 0 |

OR Odds ratio; CI confidence interval; aic Akaike's Information Criterion

**Table S4.** PEI Q2 directly after the primary care doctor appointment. Effect of the intervention bolded.

| Level | Term | Model | Estimate | Standard error | Statistic | P value | CI low | CI high | R2 nagel kerke | Aic crude | Aic adjusted | Missing data |
| --- | --- | --- | --- | --- | --- | --- | --- | --- | --- | --- | --- | --- |
| Much better | (Intercept) | crude | 2,056 | 0,166 | 4,343 | 0,000 | 1,485 | 2,845 | 0,083 | 673 |  | 12 |
| **Much better** | **Group Control** | **crude** | **0,427** | **0,238** | **-3,574** | **0,000** | **0,267** | **0,681** | **0,083** | **673** |  | **12** |
| Same or less | (Intercept) | crude | 0,241 | 0,309 | -4,609 | 0,000 | 0,131 | 0,441 | 0,083 | 673 |  | 12 |
| **Same or less** | **Group Control** | **crude** | **1,992** | **0,371** | **1,857** | **0,063** | **0,962** | **4,122** | **0,083** | **673** |  | **12** |
| Much better | (Intercept) | adjusted | 7,406 | 0,603 | 3,321 | 0,001 | 2,272 | ##### | 0,193 |  | 663 | 12 |
| **Much better** | **Group Control** | **adjusted** | **0,412** | **0,252** | **-3,521** | **0,000** | **0,251** | **0,675** | **0,193** |  | **663** | **12** |
| Much better | Age | adjusted | 0,985 | 0,008 | -1,859 | 0,063 | 0,970 | 1,001 | 0,193 |  | 663 | 12 |
| Much better | Non-Smoking | adjusted | 0,637 | 0,409 | -1,101 | 0,271 | 0,286 | 1,421 | 0,193 |  | 663 | 12 |
| Much better | Ex-Smoking | adjusted | 0,714 | 0,461 | -0,731 | 0,465 | 0,289 | 1,763 | 0,193 |  | 663 | 12 |
| Much better | Smoking | adjusted | 0,612 | 0,512 | -0,961 | 0,336 | 0,224 | 1,667 | 0,193 |  | 663 | 12 |
| Much better | Previous skin diseases Yes | adjusted | 1,009 | 0,261 | 0,035 | 0,972 | 0,605 | 1,684 | 0,193 |  | 663 | 12 |
| Much better | Multimorbidity Yes | adjusted | 0,800 | 0,281 | -0,796 | 0,426 | 0,461 | 1,386 | 0,193 |  | 663 | 12 |
| Much better | Sex Female | adjusted | 1,124 | 0,268 | 0,437 | 0,662 | 0,665 | 1,902 | 0,193 |  | 663 | 12 |
| Same or less | (Intercept) | adjusted | 0,122 | 1,060 | -1,986 | 0,047 | 0,015 | 0,973 | 0,193 |  | 663 | 12 |
| **Same or less** | **Group Control** | **adjusted** | **2,090** | **0,391** | **1,886** | **0,059** | **0,972** | **4,497** | **0,193** |  | **663** | **12** |
| Same or less | Age | adjusted | 0,997 | 0,012 | -0,229 | 0,819 | 0,974 | 1,021 | 0,193 |  | 663 | 12 |
| Same or less | Non-Smoking | adjusted | 1,659 | 0,823 | 0,615 | 0,538 | 0,331 | 8,329 | 0,193 |  | 663 | 12 |
| Same or less | Ex-Smoking | adjusted | 1,435 | 0,872 | 0,415 | 0,678 | 0,260 | 7,923 | 0,193 |  | 663 | 12 |
| Same or less | Smoking | adjusted | 0,931 | 0,983 | -0,073 | 0,942 | 0,136 | 6,386 | 0,193 |  | 663 | 12 |
| Same or less | Previous skin diseases Yes | adjusted | 0,499 | 0,402 | -1,732 | 0,083 | 0,227 | 1,096 | 0,193 |  | 663 | 12 |
| Same or less | Multimorbidity Yes | adjusted | 3,479 | 0,438 | 2,846 | 0,004 | 1,474 | 8,209 | 0,193 |  | 663 | 12 |
| Same or less | Sex Female | adjusted | 0,836 | 0,378 | -0,472 | 0,637 | 0,398 | 1,756 | 0,193 |  | 663 | 12 |

CI confidence interval; Aic Akaike's Information Criterion

**Table S5.** NPS directly after the primary care doctor appointment. Missing data: 100 patients.

| Level | Term | Model | Estimate | Standard error | Statistic | P value | CI low | CI high | R2 nagel kerke | Aic crude | Aic adjusted |
| --- | --- | --- | --- | --- | --- | --- | --- | --- | --- | --- | --- |
| Detractors | Intercept | crude | 0,143 | 0,756 | -2,575 | 0,010 | 0,032 | 0,628 | 0,045 | 254,5 |  |
| Detractors | Group: Control | crude | 1,474 | 0,935 | 0,415 | 0,678 | 0,236 | 9,210 | 0,045 | 254,5 |  |
| Promoters | Intercept | crude | 10,212 | 0,280 | 8,298 | 0,000 | 5,899 | 17,68 | 0,045 | 254,5 |  |
| Promoters | Group: Control | crude | 0,412 | 0,379 | -2,339 | 0,019 | 0,196 | 0,866 | 0,045 | 254,5 |  |
| Detractors | (Intercept) | adjusted | 0,006 | 1,085 | -4,776 | 0,000 | 0,001 | 0,047 | 0,161 |  | 262,5 |
| **Detractors** | **Group: Control** | **adjusted** | **1,145** | **0,996** | **0,136** | **0,892** | **0,163** | **8,054** | **0,161** |  | **262,5** |
| Detractors | Age | adjusted | 0,964 | 0,029 | -1,267 | 0,205 | 0,910 | 1,020 | 0,161 |  | 262,5 |
| Detractors | Non-Smoking | adjusted | 0,091 | 1,667 | -1,439 | 0,150 | 0,003 | 2,385 | 0,161 |  | 262,5 |
| Detractors | Ex-Smoking | adjusted | 0,135 | 1,852 | -1,083 | 0,279 | 0,004 | 5,075 | 0,161 |  | 262,5 |
| Detractors | Smoking | adjusted | 0,278 | 1,741 | -0,735 | 0,462 | 0,009 | 8,432 | 0,161 |  | 262,5 |
| Detractors | Previous skin diseases: Yes | adjusted | 0,682 | 1,017 | -0,377 | 0,706 | 0,093 | 4,999 | 0,161 |  | 262,5 |
| Detractors | Multimorbidity: Yes | adjusted | 0,421 | 1,227 | -0,705 | 0,481 | 0,038 | 4,662 | 0,161 |  | 262,5 |
| Detractors | Sex: Female | adjusted | 2744,5 | 1,09 | 7,25 | 0,00 | 322,4 | 23363 | 0,161 |  | 262,5 |
| Promoters | Intercept | adjusted | 51,610 | 1,299 | 3,035 | 0,002 | 4,043 | 658,8 | 0,161 |  | 262,5 |
| **Promoters** | **Group: Control** | **adjusted** | **0,368** | **0,409** | **-2,444** | **0,015** | **0,165** | **0,820** | **0,161** |  | **262,5** |
| Promoters | Age | adjusted | 0,999 | 0,013 | -0,041 | 0,967 | 0,975 | 1,024 | 0,161 |  | 262,5 |
| Promoters | Smoking | adjusted | 0,317 | 1,077 | -1,066 | 0,286 | 0,038 | 2,619 | 0,161 |  | 262,5 |
| Promoters | Non-Smoking | adjusted | 0,264 | 1,112 | -1,199 | 0,230 | 0,030 | 2,329 | 0,161 |  | 262,5 |
| Promoters | Ex-Smoking | adjusted | 0,177 | 1,146 | -1,508 | 0,131 | 0,019 | 1,678 | 0,161 |  | 262,5 |
| Promoters | Previous skin diseases: Yes | adjusted | 0,449 | 0,417 | -1,917 | 0,055 | 0,198 | 1,018 | 0,161 |  | 262,5 |
| Promoters | Multimorbidity: Yes | adjusted | 0,892 | 0,433 | -0,264 | 0,791 | 0,382 | 2,084 | 0,161 |  | 262,5 |
| Promoters | Sex: Female | adjusted | 1,076 | 0,443 | 0,166 | 0,868 | 0,452 | 2,563 | 0,161 |  | 262,5 |

CI confidence interval; Aic Akaike's Information Criterion

**Table S6.** Pre-intervention characteristics of the professionals. Comparison between groups.

|  | Intervention (N=24) | Control (N=14) | Total (N=38) | p value |
| --- | --- | --- | --- | --- |
| Education of the professional |  |  |  | 0.205 |
| Specialist | 4 (16.7%) | 0 (0.0%) | 4 (10.5%) |  |
| Licenciate | 5 (20.8%) | 2 (14.3%) | 7 (18.4%) |  |
| Medical student | 0 (0.0%) | 1 (7.1%) | 1 (2.6%) |  |
| Nurse | 15 (62.5%) | 11 (78.6%) | 26 (68.4%) |  |
| Other | 0 (0.0%) | 0 (0.0%) | 0 (0.0%) |  |
| Working experience |  |  |  | 0.375 |
| <3 months | 0 (0.0%) | 1 (7.1%) | 1 (2.6%) |  |
| 3–9 months | 1 (4.2%) | 1 (7.1%) | 2 (5.3%) |  |
| >9 months | 23 (95.8%) | 12 (85.7%) | 35 (92.1%) |  |
| Respondent has previously managed patients with skin disorders |  |  |  | 0.264 |
| No | 1 (4.2%) | 2 (14.3%) | 3 (7.9%) |  |
| Yes | 23 (95.8%) | 12 (85.7%) | 35 (92.1%) |  |
| Satisfaction with the care pathway |  |  |  | 0.369 |
| Very satisfied | 2 (8.7%) | 1 (7.7%) | 3 (8.3%) |  |
| Satisfied | 8 (34.8%) | 7 (53.8%) | 15 (41.7%) |  |
| Neutral | 7 (30.4%) | 5 (38.5%) | 12 (33.3%) |  |
| Unsatisfied | 5 (21.7%) | 0 (0.0%) | 5 (13.9%) |  |
| Very unsatisfied | 1 (4.3%) | 0 (0.0%) | 1 (2.8%) |  |
| NPS distribution |  |  |  | 0.125 |
| Detractors | 9 (40.9%) | 1 (10.0%) | 10 (31.2%) |  |
| Passives | 11 (50.0%) | 6 (60.0%) | 17 (53.1%) |  |
| Promoters | 2 (9.1%) | 3 (30.0%) | 5 (15.6%) |  |

**Table S7.** Post-intervention statistics of the professionals. Comparison between groups.

|  | Intervention, post (N=28) | Control, post (N=9) | Total (N=37) | p value |
| --- | --- | --- | --- | --- |
| Education of the professional |  |  |  | 0.684 |
| Specialist | 3 (10.7%) | 0 (0.0%) | 3 (8.1%) |  |
| Licentiate | 9 (32.1%) | 3 (33.3%) | 12 (32.4%) |  |
| Medical student | 0 (0.0%) | 0 (0.0%) | 0 (0.0%) |  |
| Nurse | 15 (53.6%) | 6 (66.7%) | 21 (56.8%) |  |
| Other | 1 (3.6%) | 0 (0.0%) | 1 (2.7%) |  |
| Working experience |  |  |  | 0.194 |
| <3 months | 2 (7.1%) | 0 (0.0%) | 2 (5.4%) |  |
| 3–9 months | 6 (21.4%) | 0 (0.0%) | 6 (16.2%) |  |
| >9 months | 20 (71.4%) | 9 (100.0%) | 29 (78.4%) |  |
| Respondent has previously managed patients with skin disorders |  |  |  | 0.379 |
| No | 7 (25.0%) | 1 (11.1%) | 8 (21.6%) |  |
| Yes | 21 (75.0%) | 8 (88.9%) | 29 (78.4%) |  |
| Respondent has treated patients with skin disorders during the new care pathway |  |  |  | 0.205 |
| No | 2 (7.1%) | 2 (22.2%) | 4 (10.8%) |  |
| Yes | 26 (92.9%) | 7 (77.8%) | 33 (89.2%) |  |
| Satisfaction with the care pathway |  |  |  |  |
| Very satisfied | 20 (71.4%) | 5 (55.6%) | 25 (67.6%) | 0.155 |
| Satisfied | 4 (14.3%) | 4 (44.4%) | 8 (21.6%) |  |
| Neutral | 4 (14.3%) | 0 (0.0%) | 4 (10.8%) |  |
| Unsatisfied | 0 (0.0%) | 0 (0.0%) | 0 (0.0%) |  |
| Very unsatisfied | 0 (0.0%) | 0 (0.0%) | 0 (0.0%) |  |
| The new care pathway is more useful for the patient |  |  |  | 1 |
| Yes | 26 (100.0%) | 9 (100.0%) | 35 (100.0%) |  |
| No | 0 (0.0%) | 0 (0.0%) | 0 (0.0%) |  |
| The new care pathway is less useful for the patient |  |  |  | 0.419 |
| Yes | 2 (7.7%) | 0 (0.0%) | 2 (5.9%) |  |
| No | 24 (92.3%) | 8 (100.0%) | 32 (94.1%) |  |
| The new care pathway reduces workload |  |  |  | 0.724 |
| Yes | 15 (60.0%) | 6 (66.7%) | 21 (61.8%) |  |
| No | 10 (40.0%) | 3 (33.3%) | 13 (38.2%) |  |
| The new care pathway increases workload |  |  |  | 0.090 |
| Yes | 1 (3.8%) | 2 (22.2%) | 3 (8.6%) |  |
| No | 25 (96.2%) | 7 (77.8%) | 32 (91.4%) |  |
| The new care pathway makes the management of patients with skin disorders |  |  |  | 0.701 |
| Easier | 21 (80.8%) | 7 (77.8%) | 28 (80.0%) |  |
| More complicated | 1 (3.8%) | 1 (11.1%) | 2 (5.7%) |  |
| No difference | 4 (15.4%) | 1 (11.1%) | 5 (14.3%) |  |
| I would like to continue with the new care pathway |  |  |  | 0.551 |
| Yes | 25 (96.2%) | 9 (100.0%) | 34 (97.1%) |  |
| No | 1 (3.8%) | 0 (0.0%) | 1 (2.9%) |  |
| My ability to treat skin conditions has improved |  |  |  | 0.994 |
| A lot | 3 (11.5%) | 1 (11.1%) | 4 (11.4%) |  |
| Somewhat | 11 (42.3%) | 4 (44.4%) | 15 (42.9%) |  |
| Not at all | 12 (46.2%) | 4 (44.4%) | 16 (45.7%) |  |
| NPS distribution |  |  |  | 1 |
| Detractors | 0 (0.0%) | 0 (0.0%) | 0 (0.0%) |  |
| Passives | 5 (17.9%) | 2 (22.2%) | 7 (18.9%) |  |
| Promoters | 23 (82.1%) | 7 (77.8%) | 30 (81.1%) |  |

NPS NetPromoter Score
